# Ioncopy: an R Shiny app to call copy number alterations in targeted NGS data

**DOI:** 10.1186/s12859-018-2159-5

**Published:** 2018-04-24

**Authors:** Jan Budczies, Nicole Pfarr, Eva Romanovsky, Volker Endris, Albrecht Stenzinger, Carsten Denkert

**Affiliations:** 1 Institute of Pathology, Charité Universitätsmedizin Berlin, Berlin, Germany; 2 Institute of Pathology, Technical University Munich (TUM), Munich, Germany; 3 Institute of Pathology, University Hospital Heidelberg, Heidelberg, Germany; 4 German Cancer Consortium (DKTK), Berlin, Munich and Heidelberg partner sites, Germany

**Keywords:** Copy number alterations, NGS, Amplicon sequencing, R Shiny

## Abstract

**Background:**

Somatic copy number alterations (CNAs) contribute to the clinically targetable aberrations in the tumor genome. For both routine diagnostics and biomarkers research, CNA analysis in a single assay together with somatic mutations is highly desirable.

**Results:**

Ioncopy is a validated method and easy-to-use software for CNA calling from targeted NGS data. Copy number and significance of CNA are estimated for each gene in each sample. Copy number gains and losses are called after multiple testing corrections controlling FWER or FDR.

**Conclusions:**

Ioncopy facilitates calling of CNAs in a cohort of tumors tissues with or without using normal (germline) DNA controls.

**Electronic supplementary material:**

The online version of this article (10.1186/s12859-018-2159-5) contains supplementary material, which is available to authorized users.

## Background

In addition to conventional somatic mutations such as point mutations and small indels, clinically relevant genetic alterations in tumors include large-scale somatic aberrations such as translocations and copy number alterations (CNAs). As classical example of an actionable CNA, testing for *HER2* amplifications revolutionized breast cancer care, since the FDA approved Herceptin for the treatment of metastatic and later for early breast cancer [1]. Both the numbers of patients and genes commonly tested for amplifications in routine diagnostics keep continuously growing. For example, amplified *HER2*, *MET* and *FGFR1* are potential drug targets in lung and other cancers [2–4]. *MDM2* is used in diagnostics of liposarcoma [5] and has recently been described to be associated with hyperprogression of metastatic cancer after immune therapy [6].

Gene amplifications are usually tested using in situ hybridization (ISH) based assays. On the other hand, targeted NGS, e.g. using the IonTorrent S5 or the Illumina MiSeq platform, is the today’s mainstay for routine testing of mutations. Therefore, integrating mutation analysis and CNA detection in a single assay would be highly desirable for both routine diagnostics and biomarker research.

Calling of CNAs from targeted sequencing data has shown to be feasible by several authors after publication of the first larger study [7]. Sensitivities obtained for *HER2* amplification calling in breast cancer range between 89% and 93% at specificities between 98% and 100% [8–10]. Methodically, all approaches use DNA sequencing coverages as input and rely on calling coverage outliers after data normalization. Most of the current algorithm requires sequencing of paired tumor and normal (germline) DNA samples [11] or utilize a normal DNA references for normalization [10, 12, 13]. Outlier detection relies on simple thresholding [7, 9, 14] or more sophisticated methods of *p*-value estimation [8, 10] or bootstrap based estimation of confidence intervals [15].

Ioncopy is a method for calling of CNAs from targeted NGS data validated before [8], which can be run in two modes either using only the cohort of interest as input (and outlier detection by internal statistics) or making use of an additional reference cohort (e.g. of normal tissue or blood samples). The extended version presented here includes a GUI for easy data upload and straightforward CNA analysis.

## Implementation

Ioncopy is an R Shiny app that can be operated without knowledge of the R programming language [16]. Input file format, analysis parameters and the formats of the output files are described in the user manual (Additional file 1).

Amplicon coverages of the cohort of interest (and optionally additionally of a reference cohort) are used as input. Coverage data are uploaded as one or several tab-separated files. Data procession and CNA calling are performed as follows: 1. Sample normalization: Each sample is scaled with the median of its amplicon coverages. 2. Amplicon normalization: If reference coverages are available, each amplicon is scaled by its median coverage in the reference data. If no reference coverages are available, each amplicon is scaled by its median coverage in the target data. Multiplication by two (corresponding to two alleles) is performed to obtain estimates of copy numbers (CNs). 3. Significance assessment for CNAs in each amplicon and each sample: First, a normal distribution centered around CN = 2 with variance estimated from the median average deviation (mad) is fitted to the distribution of CNs for each amplicon. Then, a *p*-value is calculated for each amplicon and each sample assessing the degree of being an outlier to normal distribution. 4. In the mode “gene-wise”, the *p*-values of all amplicons interrogating a gene are summarized to a single p-value using Fisher’s method [17] and CNs are summarized by taking the amplicon average. 5. Either, no multiple testing corrections, multiple testing corrections with respect to samples, multiple testing corrections with respect to genes or multiple testing corrections with respect to samples and genes are done. Then, *p*-values are corrected to control either family-wise error rate (FWER) or false discovery rate (FDR) using the Bonferroni or the Benjamini-Hochberg method respectively. 6. CNAs (gains and losses) are called when passing the significance threshold (corrected *p* < 0.05).

The intra-gene inconsistency (IGI) is a quality measure for a sample *s* that is calculated from its amplicon coverages CN(*a, s*) by

$$ \mathrm{IGI}(s)=\sum \limits_{g\in G}\ \sqrt{\frac{1}{n(g)-1}\sum \limits_{a\in A(g)}{\left[\mathrm{CN}\left(a,s\right)-\mathrm{CN}\left(g,s\right)\right]}^2}. $$

Therein, *G* denotes the set of all genes, *n*(*g*) the number and *A*(g) the set of amplicons interrogating the gene *g*. CN(*g, s*) is the CN estimate for gene *g* calculated as average of CN(*a, s*) over the amplicons *a* interrogating the gene *g*. Higher IGI corresponds higher intra-gene variance of CNs and is a technical issue related to inferior sample quality in the most cases. The IGI for each sample is included in the sample-centered output file of CNAs (CNAs samples: list). Hierarchical clustering in the heatmap display is performed using Manhattan distance and the average linkage method.

## Results and discussion

**Fig. 1:**
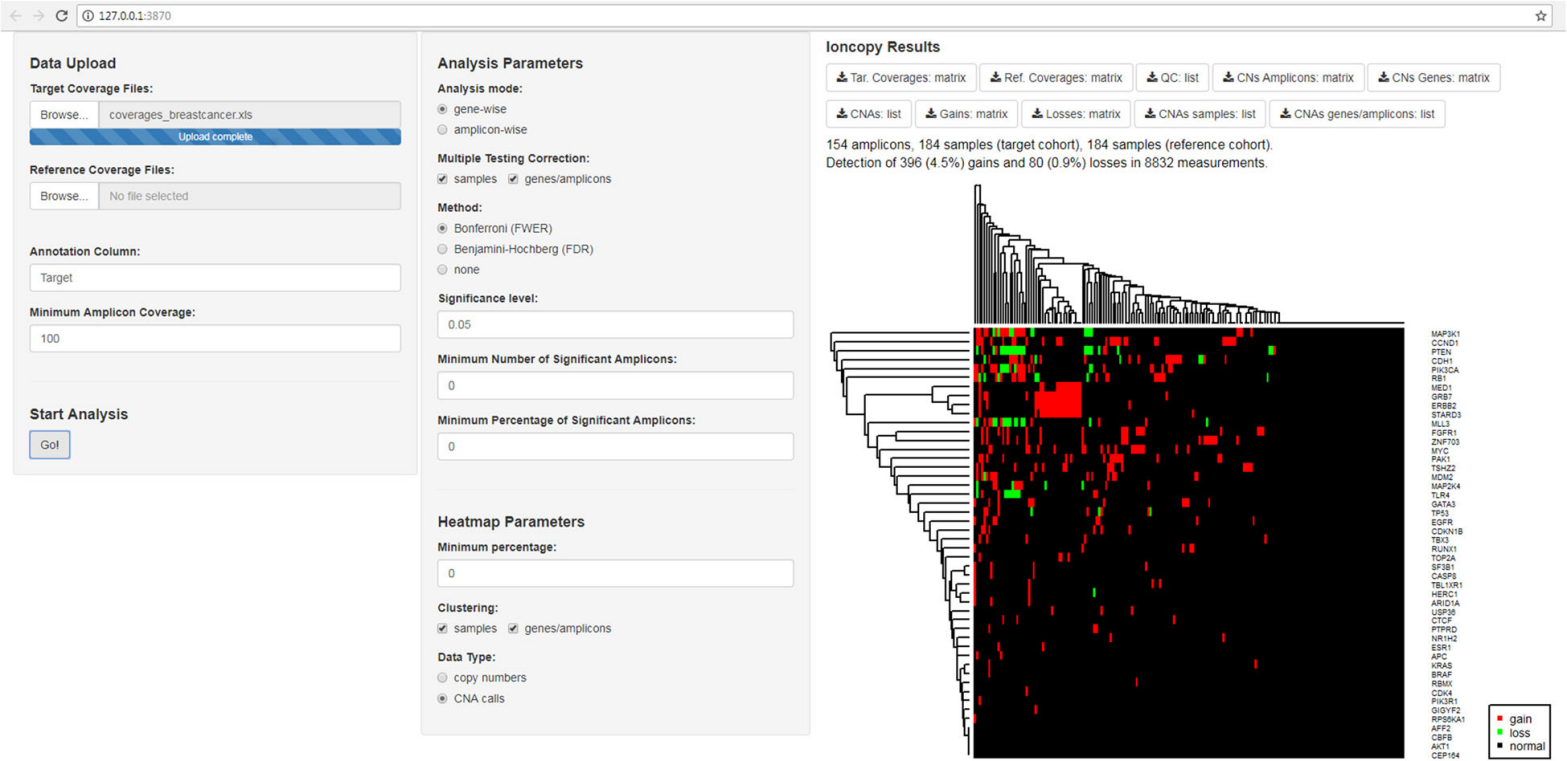
Screenshot of the Ioncopy GUI. The left panel functions to upload the matrix of sequencing coverages (amplicons × samples) and to start the analysis. In the middle panel, the user chooses gene-wise or amplicon-wise analysis and a method to correct CNA *p*-values for multiple testing. The appearance of the overview heatmap can be modified using the lower part of the middle panel. The right panel functions as result area and offers download of tables including calculated copy numbers, significances and CNA calls

As use case, we analyze 184 breast carcinoma sequenced on an Ion Torrent PGM using a 154-amplicon-panel and published before [8]. After loading of the ioncopy package and starting of the app by runIoncopy(), data upload and analysis can be performed GUI-based and without knowledge of the R language (Fig. 1). First, the coverage matrix of the breast cancer cohort (Additional file 2) is uploaded as target coverage file. Upload of a reference coverage matrix is optional and left out here. After hitting “Go!” Ioncopy estimates CNs and significances of CNAs for each gene in each sample. In the middle panel of the GUI, it can be chosen to perform the analysis either amplicon-wise or gene-wise. Multiple testing corrections and CNA calling can be performed in a more or a less stringent ways. In the gene-wise mode, the user can chose to call only CNAs that are supported by two or more calls of amplicons or by 50% (or another selectable percentage) of the amplicons located in the gene under consideration.

Operating Ioncopy in the gene-wise analysis mode, 396 (4.5%) gains and 80 losses (0.9%) are detected for FWER control at *p* = 0.05 taking into account multiple testing for both genes and samples. Out of these, 351 (4.0%) gains and 59 (0.7%) losses are supported by calls of at least two amplicons. The analysis mode of FWER control can be relaxed to FDR control at 5%, which leads to detection of 856 (9.7%) gains and 295 (3.3%) losses. Also, it is possible to run Ioncopy without multiple testing corrections, which is recommendable only for usage as search test together with validation of CNA candidates by an independent confirmatory test.

Called CNAs can be downloaded as matrix genes × samples, as gene-focused list or as sample-focused list. The latter includes IGI as a sample quality measure that is calculated from the CNs as described in the Implementation section. High IGI corresponding to inconsistency of CNs within genes and inferior coverage data quality, which is often detected in samples with low DNA quality (data not shown).

**Fig. 2:**
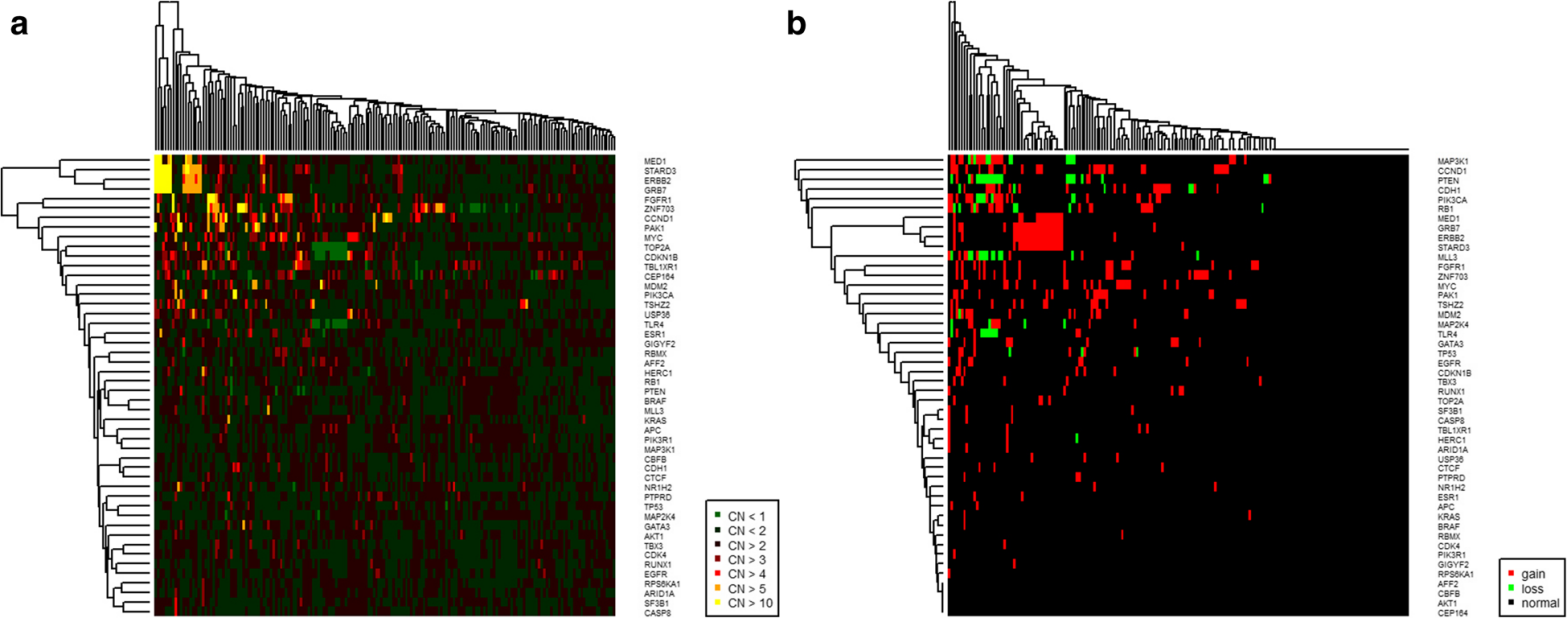
Heatmaps of CN levels (**a**) and CNA calls (**b**). In both kinds of heatmaps samples and/or genes can be either left in the order of the input data or be clustered. Sparing sample clustering can be helpful for technical quality control and exclusion of batch effects

Heatmaps are generated to display the estimated CNs or the detected CNAs (Fig. 2). In the breast cancer data, *ERBB2*, *GRB7*, *STARD3* and *MED1*, all located in the chromosomal region 17q12 and typically co-amplified in HER2+ breast cancer, cluster tightly together. Several options are available to influence the appearance of the heatmaps. For example, sample clustering can be spared to preserve the natural order of the input data what can be helpful for technical quality control and exclusion of batch effects.

A limitation of NGS based CNA detection is tumor purity, as detection sensitivity decreases significantly when the tumor cell content falls below 50% [8]. For low tumor content samples, spatial-dissolved assays such as FISH should be preferred for CNA detection instead of bulk tissue based detection methods.

## Conclusions

Ioncopy is a freely available and easy-to-use method for calling CNAs from targeted NGS data.

## Availability and requirements

**Project name:** Ioncopy.

**Project home page:** https://cran.r-project.org/package=ioncopy

**Operating system:** Platform independent.

**Programming language:** R Shiny.

**License:** GNU GPL 3.

**Restrictions to use by non-academics:** none.

## Additional files

Additional file 1: Ioncopy user manual. Comprehensive description of the input file format, of the analysis parameters and of the format of the output files. (PDF 1690 kb)

Additional file 2: Breast cancer example data set. Coverage matrix of 152 amplicons (48 genes) in 184 breast cancer samples. (XLS 196 kb)

## Abbreviations

CN: Copy number
CNA: Copy number alteration
FDR: False discovery rate
FWER: Family-wise error rate
IGI: Intra-gene inconsistency
