# Supplementary material for: Ioncopy: an R Shiny app to call copy number alterations in targeted NGS data
Source: BMC Bioinformatics. 2018 Apr 24;19:157. doi: 10.1186/s12859-018-2159-5 (PMC5921540; doi:10.1186/s12859-018-2159-5)
Supplement: Supplementary file 1 — Ioncopy user manual. Comprehensive description of the input file format, of the analysis parameters and of the format of the output files. (PDF 1690 kb) [file 12859_2018_2159_MOESM1_ESM.pdf]

Ioncopy (v2.1) user manual

Jan Budczies

March 23, 2018

Contents

1 Introduction **2**

2 Data upload **2**

3 Estimation of copy numbers **3**

4 Significance assessment **4**

5 Gene-wise analysis **4**

6 Multiple testing correction **4**

7 Filtering for validated CNAs **4**

8 Data analysis **4**

9 Heatmap **5**

10 Quality assessment **5**

11 Data download **6**

1 Introduction

Ioncopy is a method for calling copy number alterations (CNAs) in amplicon sequencing data including based outlier dection in coverage data. This manual describes how to use the graphical user interface (GUI) for package version 2.1 that is implemented as Shiny app. After installation of ioncopy (available from the CRAN repositoy) the app can be launched by:

```
R> library(ioncopy)
R> runIoncopy()
```

These are the most important steps to run an Ioncopy CNA analysis (Fig. 1):

1. Upload of target coverage data and (optionally) reference coverage data
2. Selection of the analysis mode (gene-wise or amplicon-wise)
3. Selection of the the method for multiple testing
4. Optionally: Select a minimum number of significant amplicons and/or a minimum percentage of significant amplicons
5. Hit "Go!" to start the analysis
6. Change of the heatmap parameters (optionally)
7. Download of the lists of called copy number gains and losses

2 Data upload

An example coverage file for upload (coverages_breastcancer.xls) can be found in the inst/extdata subdirectory of the package. Coverage data of target cohort and optionally reference cohort can be uploaded from a single or from multiple files. All coverges files have to belong to the same sequencing panel. If no file is being seleced for the reference cohort, the data of the target cohort are used as reference data. The coverage data should be prepared as tab-separated spread sheets with the amplicons in the row in the samples in the columns. This can be obtained by saving an Exel spread sheet as text (.txt) file. Each of the colums should have a column name. All colums containing numeric data are interpreted as coverages of the sample decribed by the column name. One of the columns that contain non-numeric data should contain the names of the amplicons. This column needs to be defined as annotation column in the GUI. The names of the amplicons should be of

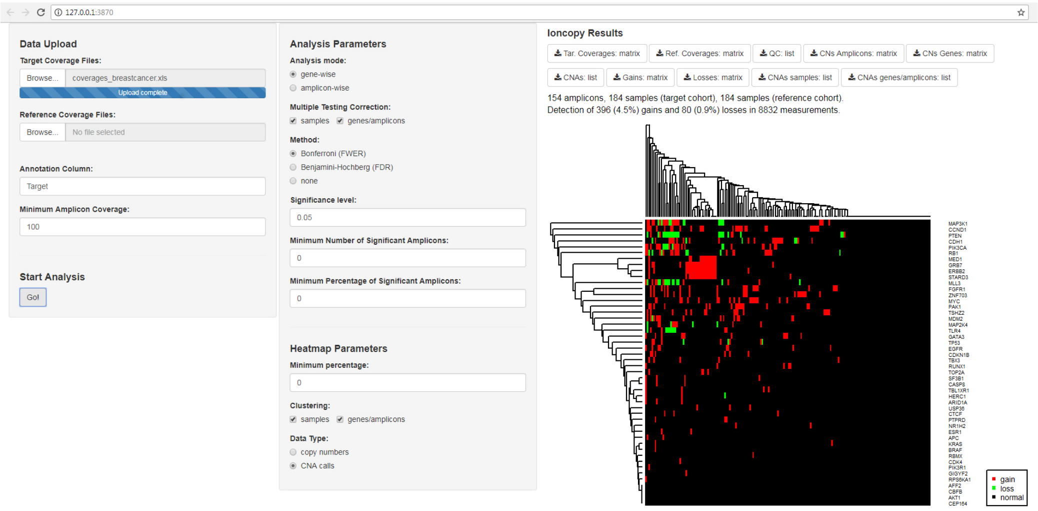

Figure 1: Ioncopy GUI. Upload of target coverage data and (optionally) reference coverage data (left panel). Seletion of analysis parameters (middle panel). Download of result files and heatmap visualization (right panel)

the form `<gene>_<amplicon>` where `<gene>` is an unique identifier referring to the gene and `<amplicon>` is a unique identifier referring to the amplicon. It is imporant to comply with this nomenclature to enable Ioncopy to identify the amplicons that belong to a gene. It is possible to perform a quality filtering by including only the amplicons with a minimum mean coverage in the reference cohort into the analysis. This can be done by entering a non-zero value in the field "minimum amplicon coverage" (default value: 100).

3 Estimation of copy numbers

Coverage data are processed by a twofold normalization: 1. Each sample is scaled with the median of its amplicon coverages (sample normalization). 2. If reference coverages are available, each amplicon is scaled by the median coverage of the amplicon in the reference data. If no reference coverages are available, each amplicon is scaled by its median coverage (amplicon normalization). Multiplication by two (corresponding to two alleles) is performed to obtain estimates of copy numbers (CNs).

4 Significance assessment

The significance of a potential CNA is assessed in each amplicon and each sample using parametric statistics. To this end, a normal distribution centered around CN=2 with variance estimated from the median average deviation (mad) is fitted to the distribution of CNs for each amplicon. Then, a p-value is calculated for each amplicon and each sample assessing the degree of being an outlier to the normal distribution.

5 Gene-wise analysis

The analysis can be run either in the "amplicon-wise" or in the "gene-wise" mode. In the mode of gene-wise analysis, the p-values of all amplicons of interrogating the gene are summarized to a single p-value using Fisher's method and CNs are summarized by taking the average over the amplicons. Details of the mapping of the coverage data to genes are decribed in the section "data upload".

6 Multiple testing correction

Either, no multiple testing corrections, multiple testing corrections with respect to samples, multiple testing corrections with respect to genes or multiple testing corrections with respect to samples and genes are done. It can be chosen to control either familiy-wise error rate (FWER) using the Bonferroni method or false discoveray rate (FDR) using the Benjamini-Hochberg method.

7 Filtering for validated CNAs

In the analysis mode gene-wise, the user can select to call only CNAs that are supported by more than one amplicon. There are two options available: Either, the minumum number of amplicons that need to support the call of a gene CNA is selected, and/or the minimum percentage of amplicons of all amplicons interrogating a gene is chosen.

8 Data analysis

The CNA analysis can be started by hitting the "Go!" button. A progress bar notifies which part of the analysis is currently performed. Analyzing

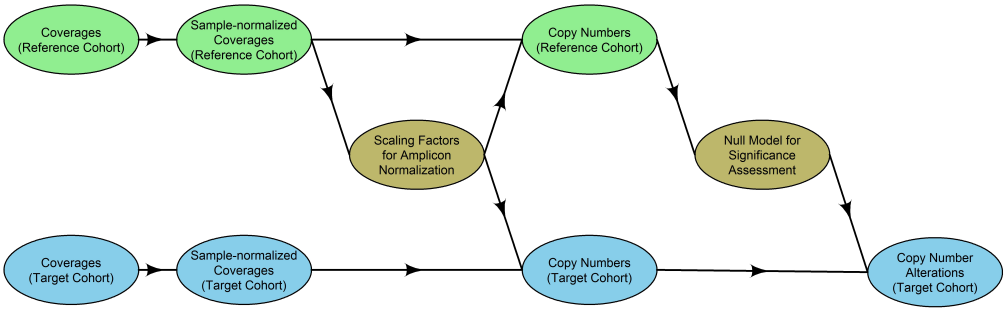

Figure 2: Workflow of Ioncopy. The target cohort comprises the DNA samples under investigation. The reference cohort comprises the DNA samples used as reference for normalization and significance assessment. Target cohort and reference cohort can be chosen to be identical.

200 sampels sequenced with a panel of 200 amplicons will take less that one minute on a common desktop PC. The worflow of the CNA analysis is shown in Fig. 2.

9 Heatmap

The genes included in the heatmap visualization can be selected by confining the diagram to the genes that are altered in a minimum percentage of samples. Hierarchical clustering of samples, genes or both can be performed. The input in the heatmap section can be modified after the CNA analysis has been finished. The heatmap output will be updated instantaneously without repeating the CNA analysis.

10 Quality assessment

Dection of CNAs relies on the reproducibility of coverage pattern between samples, i.e. this pattern should be the same in all samples without CNAs up to a factor. Based on this hypothesis CNs are estimated. CNs are expected to be constant within a gene (up to rare biological exceptions). Deviations from being constant can be measured by the intra-gene inconsistency (IGI) defined as

$$\mathrm{IGI} = \frac{1}{n} \sum_{g \in \mathrm{genes}} \sqrt{\frac{1}{n_g - 1} \sum_{a \in A_g} (\mathrm{CN}_a - \mathrm{CN}_g)^2}, \tag{1}$$

wherein n denotes the number of genes in the panel, n_g the number of amplicons interrogating gene g, A_g the set of amplicons interrogating gene g and $\mathrm{CN}_g = \frac{1}{n_g} \sum_{g \in A_g} \mathrm{CN}_a$ the copy number of gene g. Higher IGI corresponds to worse consistency and worse technical data quality. The IGI for each sample is included in the downloadable sample-centered view of CNAs (CNAs samples: list).

11 Data download

The following results can be download as Excel spread sheets:

- The coverages of the target cohort (Tar. Coverages: matrix) and the coverages of the reference cohort (Ref. Coverages: matrix).
- A list of statistical metrics for each of the of amplicons (QC: list). These include the mean coverage in the target cohort and in the reference cohort, the Shapiro-Wilk statistics and p-value (testing of the estimated CNs to be normal distributed), as well as mean, sd, median and mad of the estimated CNs.
- The estimated CNs for amplicons (CNs Amplicons: matrix) and for genes (CNs Genes: matrix).
- A list with estimated copy number, p-value and CNA call (NORMAL, GAIN or LOSS) for each pair of sample and gene/amplicon (CNAs: list).
- The gain and loss calls as binary matrices (Gains: matrix and Losses: matrix).
- The detected CNAs in a sample-centered view (CNAs samples: list) and a gene/amplicon-centered view (CNAs genes/amplicons: list).

The designation "matrix" refers to tables with genes/amplicons in the rows and samples in the columns.
